# Chloroplast redox imbalance governs phenotypic plasticity: the “grand design of photosynthesis” revisited

**DOI:** 10.3389/fpls.2012.00255

**Published:** 2012-11-20

**Authors:** Norman P. A. Hüner, Rainer Bode, Keshav Dahal, Lauren Hollis, Dominic Rosso, Marianna Krol, Alexander G. Ivanov

**Affiliations:** ^1^Department of Biology, Western UniversityLondon, ON, Canada; ^2^The Biotron Centre for Experimental Climate Change Research, Western UniversityLondon, ON, Canada

**Keywords:** acclimation, excitation pressure, phenotype, photostasis, plasticity, redox sensing/signaling

## Abstract

Sunlight, the ultimate energy source for life on our planet, enters the biosphere as a direct consequence of the evolution of photoautotrophy. Photoautotrophs must balance the light energy absorbed and trapped through extremely fast, temperature-insensitive photochemistry with energy consumed through much slower, temperature-dependent biochemistry and metabolism. The attainment of such a balance in cellular energy flow between chloroplasts, mitochondria and the cytosol is called photostasis. Photoautotrophs sense cellular energy imbalances through modulation of excitation pressure which is a measure of the relative redox state of Q_A_, the first stable quinone electron acceptor of photosystem II reaction centers. High excitation pressure constitutes a potential stress condition that can be caused either by exposure to an irradiance that exceeds the capacity of C, N, and S assimilation to utilize the electrons generated from the absorbed energy or by low temperature or any stress that decreases the capacity of the metabolic pathways downstream of photochemistry to utilize photosynthetically generated reductants. The similarities and differences in the phenotypic responses between cyanobacteria, green algae, crop plants, and variegation mutants of *Arabidopsis thaliana* as a function of cold acclimation and photoacclimation are reconciled in terms of differential responses to excitation pressure and the predisposition of photoautotrophs to maintain photostasis. The various acclimation strategies associated with green algae and cyanobacteria versus winter cereals and *A. thaliana* are discussed in terms of retrograde regulation and the “grand design of photosynthesis” originally proposed by Arnon (1982).

## INTRODUCTION

**FIGURE 1:**
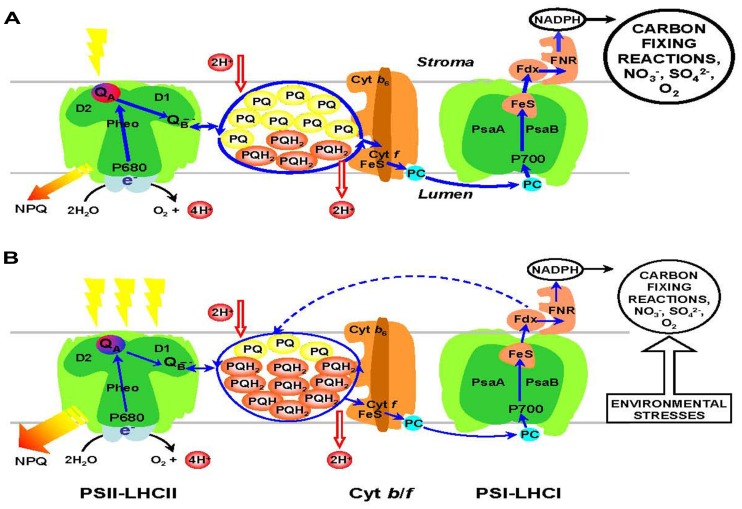
**General model of the photosynthetic electron transport chain. (A)** Low excitation pressure conditions are indicated by a single yellow lighting bolt. Under these conditions, the PQ pool is largely in the oxidized state because the rate of photosynthetic electron transport plus NPQ match the rate of consumption of photosynthetic reductant, NADPH. Thick solid blue arrows indicate linear electron transport from photosystem II (PSII) to photosystem I (PSI) to reduce NADP^+^ to NADPH which is consumed by C, N, and S metabolism. Energy dissipated as heat through NPQ is minimal. PQ, plastoquinone (yellow); PQH_2_, plastoquinol (orange). **(B)** High excitation pressure conditions can be generated by either high light (3 lightning bolts) or by environmental stresses such as low temperature or nutrient stress which inhibit rates of metabolism. Under these conditions, the PQ pool is largely reduced to plastoquinol and energy dissipated through NPQ is increased. Consequently, rates of linear electron transport between PSII and PSI decrease (thin solid blue arrows) and enhance rates of PSI cyclic electron transport (broken blue arrow).

Evolution has harnessed sunlight as the energy source for life because it is cheap, abundant, available in a very predictable manner and present in seemingly inexhaustible quantities when measured on a biological time scale. In photoautotrophic eukaryotes, the integral, thylakoid membrane, chlorophyll-pigment-protein complexes associated with photosystem II (PSII) and photosystem I (PSI) absorb, convert, and trap this energy as electrons (**Figure 1**). Intersystem photosynthetic electron transport (PET) connects the two photosystems through coupled oxidation–reduction of the plastoquinone (PQ) pool, the Cytochrome b_6_/f complex (Cyt b_6_/f), and plastocyanin (PC). The net result of linear PET is the biosynthesis of reducing power in the form of NADPH and chemical energy in the form of ATP. Alternatively, electrons generated by PSI can be re-cycled through PSI cyclic PET which allows photosynthetic organisms to regulate ATP/NADPH ratios in the chloroplast (Shikanai, 2007; Johnson, 2011)

An astonishing characteristic of photosynthesis is that evolution has combined processes that exhibit extreme disparities in temperature sensitivities and rate constants that differ by at least 10 orders of magnitude (Hüner and Grodzinski, 2011). Consequently, photosynthetic organisms are predisposed to maintain a balance between the rates of light energy trapping through extremely fast (femtosecond to picosecond time scale) but temperature-insensitive photophysical and photochemical processes of light absorption, energy transfer, and charge separation that generates electrons within the photosynthetic reaction centers versus the much slower but very temperature-sensitive processes of C, N, and S-metabolism (**Figure 1**), and ultimately growth and development that utilize the photosynthetic reductants. To overcome this disparity in reaction rates and temperature sensitivity, non-photochemical quenching mechanisms (NPQ) have evolved to dissipate any excess energy not used in photosynthesis as heat either through antenna quenching via the xanthophyll cycle (Demmig-Adams and Adams, 1992; Horton et al., 1996, 2008; Demmig-Adams et al., 1999) and/or reaction center quenching through PSII charge recombination (Krause and Weis, 1991; Walters and Horton, 1993; Hüner et al., 2006) to protect the PSII reaction center from over-excitation and ensure survival in a fluctuating light environment (**Figure 1**). The balance between energy trapping versus energy utilization and/or dissipation is called photostasis.

Photostasis can be represented by the equation, σ_PSII_·*E*_k_ = τ ^−1^ (Falkowski and Chen, 2003; Hüner et al., 2003) where σ_PSII_ is the effective absorption cross-section of PSII, *E*_k_ is the irradiance (I) at which the maximum photosynthetic quantum yield balances photosynthetic capacity and τ ^−1^ is the turnover rate of metabolic sinks, such as the assimilation of C, N, or S and ultimately by growth and development, that consume photosynthetic electrons. The product σ_PSII_·*E*_k_ is, by and large, insensitive to temperature in the biologically significant range (0–45°C) because it reflects the photophysical processes of light absorption and energy transfer within the light-harvesting antennae and core antennae pigment–protein complexes, which result in the induction of photochemistry and a charge separated state in the PSII reaction center. Although PSI absorbs light, its photochemical turnover rate is much higher than PSII and is normally not considered to be rate-limiting in PET (Ke, 2001) and therefore is not included in the equation for photostasis. In contrast, τ ^−1^, which reflects biochemical reactions that consume photosynthetically generated electrons, is very temperature-sensitive (Melis, 1998; Hüner et al., 2003; Ensminger et al., 2006; Wilson et al., 2006; McDonald et al., 2011).

High excitation pressure (HEP) is a consequence of an imbalance between energy trapped through photochemistry versus energy either utilized through biochemistry or dissipated through NPQ and will occur whenever σ_PSII_·*E*_k_ > τ ^−1^ (Hüner et al., 2003; Ensminger et al., 2006; Wilson et al., 2006). Since the oxidation of plastoquinol (PQH_2_) by the Cyt b_6_/f complex is the rate-limiting step of intersystem PET (Haehnel, 1984), HEP results in an over-reduction of the PQ pool and the intersystem PET chain (**Figure 1**). This can be detected *in vivo* as an accumulation of closed PSII reaction centers and quantified by Chl a fluorescence as either 1-qP (Dietz et al., 1985; Hüner et al., 1998) or 1-qL (Hendrickson et al., 2004; Kramer et al., 2004; Baker, 2008) where qP and qL represent the photochemical quenching parameter (Schreiber et al., 1994; Baker, 2008). HEP occurs under any condition whereby *I* > *E*_k_, and can be generated by many environmental conditions including exposure to high light (HL) or any combination of HL or low temperature (LT), nutrient limitation or water status. Thus, theoretically, the effects of acclimation to any of these stresses on the structure and function of the photosynthetic apparatus should mimic acclimation to high irradiance (Melis, 1998; Hüner et al., 2003; Ensminger et al., 2006; Wilson et al., 2006).

Photoautotrophs respond to growth and development under HEP by remodeling the structure and function of the photosynthetic apparatus to balance cellular energy flow and establish a new photostatic state. According to the equation for photostasis, this can occur in the following ways: first, by enhancing sink capacity (τ ^−1^) through increased rates of energetically “useful” processes that consume photosynthetic reductants and fixed C such as respiration, N-assimilation and ultimately growth which results in increased biomass production. Alternatively, photostasis can be achieved by decreasing the efficiency of light absorption and trapping (σ_PSII_) through energetically “wasteful” processes by either increasing rates of NPQ through stimulation of the xanthophyll cycle in the short-term (Demmig-Adams and Adams, 1992; Demmig-Adams et al., 1999) and/or reducing the physical size of the light-harvesting complex to decrease the probability of light absorption itself in the long-term (Hüner et al., 2003). However, the specific strategies employed to re-establish photostasis appear to be species-dependent and may result in alterations in the observable characteristics of an individual due to genotype versus environment interactions. Thus, a single genotype may exhibit variable phenotypes in response to changes in their environment. This is defined as phenotypic plasticity which reflects the integrated regulation of transcriptional, translational, and post-translational events with higher order processes associated with metabolism, growth, and photomorphogenesis. The latter is defined as the development of plant form and structure by light other than that utilized in photosynthesis. In contrast to photosynthesis, photomorphogenesis is regulated by specialized photoreceptors such as phytochrome and cryptochrome (Quail et al., 1995; Cashmore, 1997; Whitelam and Devlin, 1998). The focus of this review is on the role of light absorbed by the photosynthetic apparatus in governing phenotypic plasticity independent of photomorphogenesis. Fey et al. (2005) showed that redox signals from the photosynthetic apparatus can operate through retrograde signaling to affect nuclear gene expression independently of signaling through photoreceptors involved in photomorphogenesis. Furthermore, using various photomorphogenic mutants of *Arabidopsis thaliana*, Walters et al. (1999) showed that *Arabidopsis* mutants impaired in photomorphogenesis still retained the ability to adjust the structure and function of the photosynthetic apparatus in response to changes in growth irradiance. Thus, photoreceptors involved in photomorphogenesis are not required for the remodeling of the photosynthetic apparatus during the re-establishment of photostasis.

Energy sensing/signaling, retrograde regulation, and the molecular mechanisms that underlie phenotypic plasticity are complex, integrated cellular processes. Due to inherent restrictions with respect to length of this manuscript, we are not able to provide an exhaustive review of all pertinent published data in these areas of research. Rather, we focus on specific examples of acclimation to irradiance and temperature to illustrate how excitation pressure sensed within the chloroplast governs both local as well as distant molecular events to affect phenotypic plasticity.

## REGULATION OF PHENOTYPIC PLASTICITY IN GREEN ALGAE AND CYANOBACTERIA

Growth and development of the green algae, *Dunaliella tertiolecta*, *D. salina*, and *Chlorella vulgaris*, under HL results in a typical yellow to yellow-green, HEP phenotype which is characterized by low Chl per cell and high ratios of Chl a/b (≥10) compared to the typical green phenotype observed upon growth at low light (Sukenik et al., 1987; Escoubas et al., 1995; Maxwell et al., 1995a,b; Wilson and Hüner et al., 2000). Since the nuclear encoded LHCII polypeptides bind the bulk of the Chl in eukaryotic chloroplasts (Green et al., 2003), this HL phenotype reflects alterations in σ_PSII_. Since the equivalent phenotype is generated by growth at LT and moderate irradiance which generates an excitation pressure comparable to the HL condition in *D. salina* and *Chlorella vulgaris* as well as the filamentous cyanobacterium, *Plectonema boryanum*, this phenotype is not a HL phenotype *per se* but rather should be considered a HEP phenotype (Maxwell et al., 1995a,b; Hüner et al., 1998, 2003; Miskiewicz et al., 2000, 2002; Wilson et al., 2003, 2006; Ensminger et al., 2006). The decrease in σ_PSII_ in response to growth at HEP can be reconciled, in part, by the fact that *Chlorella vulgaris* exhibits a limited capacity to adjust photosynthetic carbon metabolism (Savitch et al., 1996) and growth rates (Wilson and Hüner et al., 2000) in response to HL. Similar results have been reported for *P. boryanum* (Miskiewicz et al., 2000, 2002). Thus, neither of these photosynthetic microbes is able to adjust their sink capacity (τ ^−1^) sufficiently to balance the increased energy input due to HL. Thus, to survive under HL conditions, these organisms decrease σ_PSII_ by decreasing their efficiencies to harvest and trap light coupled with enhanced dissipation of absorbed excess light through NPQ. Similarly, LT inhibits growth due to thermodynamic constraints and to survive at LT, these organisms also decrease σ_PSII_ to compensate for the lower growth rates thus lower sink capacity (τ ^−1^) for a given irradiance (Miskiewicz et al., 2000; Wilson and Hüner et al., 2000). Thus, the phenotypic congruence between acclimation to either LT or HL in these unicellular organisms appears to be a consequence of limitations in their capacity to adjust sink capacity in response to changes in temperature and light which generates a comparable HEP condition.

Complementary chromatic adaptation (CCA) is a phenotypic change exhibited by cyanobacteria in response to changes in ambient light quality (Kehoe and Gutu, 2006; Gutu and Kehoe, 2012). CCA is an historical misnomer and is actually an acclimation response to changes in light color (Kehoe and Gutu, 2006). When the filamentous cyanobacterium, *Fremyella diplosiphon*, is grown under green light, this cyanobacterium exhibits a red pigmented phenotype whereas it exhibits a blue-green phenotype when grown under red light. This acclimation response to light color is completely reversible and reflects alterations in the major light-harvesting pigments, phycoerythrin and phycocyanin, associated with phycobilisomes (Gantt, 1994). The regulation of CCA appears to involve the integration of a phytochrome-type photoreceptor pathway that is sensitive to green and red light in addition to a pathway that is redox-sensitive and involves PET (Kehoe and Gutu, 2006).

Coordinated regulation of Chl biosynthesis, *Lhcb* transcript abundance as well as Lhcb polypeptide accumulation is likely an important characteristic of acclimation to HEP in green algae. However, retrograde regulation examined in a *gun4* mutant of *Chlamydomonas reinhardtii* indicates that down-regulation of *LHC* genes is governed post-transcriptionally with minimal transcriptional co-ordination (Formighieri et al., 2012). Chl b is required for the assembly and stabilization of LHCII (Thornber et al., 1994). Masuda et al. (2003) demonstrated that changes in the levels of *CAO* transcripts, encoding the enzyme catalyzing the conversion of Chl a to Chl b, occur concomitantly with changes in *Lhcb* transcript abundance during acclimation to HL intensity in *D. salina *while the use of site specific inhibitors of the PET demonstrated that the redox state of the PQ pool regulates both *CAO* and *Lhcb* transcript abundance. This is consistent with previous work demonstrating the regulation of *Lhcb* transcription by the redox state of the PQ pool in *D. tertiolecta *during photoacclimation (Escoubas et al., 1995). These studies are consistent with acclimation to HEP in green algae since HL has the potential to create imbalances in energy flow (Hüner et al., 1998; Ensminger et al., 2006). The pale yellow-green pigmentation of algal cultures acclimated to HEP may reflect limitations at the level of Chl biosynthesis. Studies in *CAO* over-expressors in higher plants have indicated that changes in *CAO* transcription rates are sufficient to cause increases in the abundance of Lhcb polypeptides (Tanaka et al., 2001; Tanaka and Tanaka, 2007; Biswal et al., 2012) indicating that modulation of σ_PSII_ may occur through transcriptional regulation of Chl b biosynthesis. Similarly, levels of CAO have been correlated to Chl b abundance and LHCII antenna size in *D. salina* (Masuda et al., 2002). It is currently unclear whether retrograde signals originating from the redox state of the PQ pool during acclimation to HEP directly coordinate Chl biosynthesis and LHCII abundance in green algae through transcriptional regulation of nuclear-encoded *Lhcb* and *CAO* genes in parallel or indirectly through a regulatory mechanism involving modulation of Chl b biosynthesis at the level of transcriptional control of *CAO* expression.

The mechanisms underlying the signal transduction pathways associated with retrograde regulation between the chloroplast and the nucleus in plants and green algae remain equivocal. However, recent evidence supports the role of heme, Mg protoporphyrin IX, HSP70, and HSP90 as important components in the retrograde signaling pathway (Strand et al., 2003a; von Gromoff et al., 2008; Kindgren et al., 2012). In addition, important biochemical evidence for the involvement of a protein phosphorylation cascade has been reported for *D. salina* (Escoubas et al., 1995; Masuda et al., 2003). Protein kinase inhibitors prevented the induction of *Lhcb* and *CAO* expression during acclimation to low light intensity in *D. salina* (Masuda et al., 2003). Furthermore, *cis*-acting elements in the promoter region of algal *Lhcb* genes have been identified which are likely required for the plastidic redox regulation of nuclear gene expression (Escoubas et al., 1995; Chen et al., 2004). Furthermore, primary C and N metabolic pathways between chloroplasts and mitochondria and may also represent important communication pathways between these two organelles (Raghavendra et al., 1994; Gardestrom et al., 2002; Wilson et al., 2003). For example, inhibition of respiratory electron transport resulted in an increase in excitation pressure (Wilson et al., 2003) and decreased activation of Calvin cycle enzymes (Padmasree and Raghavendra, 2001). Furthermore, HEP stimulated the expression of the mitochondrial alternative oxidase (AOX; Rosso et al., 2009).

Does the generation of the yellow, HEP phenotype in *Chlorella vulgaris* represent a threshold response to varying excitation pressure? If so, one would expect a sigmoidal response for changes in Chl content, Chl a/b ratios and Lhcb content as a function of excitation pressure. This could be interpreted to indicate that the redox sensor(s) that respond to excitation pressure act as a “molecular on–off switch,” that is, a minimum excitation pressure must be attained before nuclear encoded *Lhcb* genes are repressed by HEP through retrograde regulation. The proxies for phenotype included total Chl/cell and Lhcb content which exhibited a linear but negative relationship with increasing excitation pressure (Maxwell et al., 1995a,b; Wilson and Hüner et al., 2000). Concomitantly, Chl a/b ratios and xanthophyll cycle activity, and hence NPQ, also varied linearly but positively, as expected, as a function of increasing excitation pressure (Wilson and Hüner et al., 2000). Since all proxies for the phenotypic response of *Chlorella vulgaris* varied linearly as a function of excitation pressure, this indicates that the redox sensor(s) that govern the phenotypic response to excitation pressure in *Chlorella vulgaris* is not a “molecular on–off switch” but rather is analogous to a “molecular rheostat.”

## ROLE OF CBF TRANSCRIPTION FACTORS IN THE REGULATION OF PHENOTYPIC PLASTICITY IN TERRESTRIAL PLANTS

*Brassica napus*, winter cereals such wheat and rye grown at LTs exhibit a developmental shift from an elongated to a dwarf growth habit (Gray et al., 1997; Dahal et al., 2012a,b). However, the biomass of the dwarf plants are equal to or higher than the plants which exhibit the elongated phenotype due to a combination of increased leaf thickness, increased cytoplasmic volume coupled with decreased water content with no change in the total number of leaves (Hüner et al., 1984; Krol et al., 1984; Boese and Hüner et al., 1990; Strand et al., 1999; Gorsuch et al., 2010; Dahal et al., 2012a,b). Previously, it was presumed that this dwarf growth habit was strictly a response to growth at LT and this phenotype was used to select for freezing tolerance (Levitt, 1980). However, growth at HL but warm temperatures generates a comparable dwarf growth habit as observed at LT. Thus, it was shown that this dwarf phenotype is, in fact, governed by excitation pressure rather than by LT (Gray et al., 1997; Hüner et al., 1998). In contrast to green algae, *Chlorella vulgaris* and *D. salina* and the cyanobacterium, *P. boryanum*, these cold acclimated winter cultivars maintain photostasis by matching a high efficiency for light absorption (σ_PSII_) with an increased capacity for CO_2_ assimilation (τ ^−1^) through the up-regulation of transcription and translation of genes coding for Rubisco and the regulatory enzymes of cytosolic sucrose and fructan biosynthesis (Savitch et al., 2000a; Stitt and Hurry, 2002; Strand et al., 2003b; Ensminger et al., 2006; Dahal et al., 2012a,b) coupled with enhanced rates for leaf carbon export (Leonardos et al., 2003) and the suppression of photorespiration (Savitch et al., 2000b). These results are consistent with the global analyses of the cold acclimated *A. thaliana* metabolome which indicate a major reprogramming of carbon metabolism relative to non-acclimated plants (Gray and Heath, 2005). As a result, energy use efficiency is enhanced because the dissipation of absorbed light energy through NPQ is kept to a minimum while absorbed light energy used for C-assimilation is maximized resulting in increased biomass accumulation (Hüner et al., 1998; Stitt and Hurry, 2002; Strand et al., 2003b; Ensminger et al., 2006; Dahal et al., 2012a,b). This not only maximizes the chemical energy stored and carbon pool available for the renewed growth and reproduction in the spring but the accumulation of photosynthetic end-products such as sucrose also provides cryoprotectants to stabilize the cell membranes against freezing events during the winter (Stitt and Hurry, 2002).

What governs this complex, integrated phenomenon which appears to involve a system-wide change in morphology, physiology, and biochemistry of cold-tolerant crop plants? It has been suggested that cold- binding transcription factors/dehydration responsive element binding factors (CBFs/DREBs) control the phenotypic plasticity and freezing tolerance in cold-tolerant species (Jaglo-Ottosen et al., 1998; Liu et al., 1998; Kasuga et al., 1999; Gilmour et al., 2000, 2004; Savitch et al., 2005; Theocharis et al., 2012). Recently, we reported that the over-expression of a specific *CBF *in *Brassica napus*, *BnCBF17*, not only induces a dwarf phenotype but concomitantly enhances photosynthetic performance, the efficiency of energy conversion, water use efficiency, and biomass production comparable to that observed in cold acclimated *B. napus *(Savitch et al., 2005; Dahal et al., 2012a). We suggest that the transcription factor, *BnCBF17*, may be either a master regulator or certainly a central component which governs the regulation of plant architecture, photosynthetic capacity, and energy conversion efficiency of crops. In *Arabidopsis*, the protein kinases, KIN10/KIN11, act as a central integrator of transcription networks associated with plant carbon metabolism and energy balance (Baena-Gonzalez et al., 2007). CBFs may interact with KIN10/KIN11 to affect photosynthetic performance in response to excitation pressure. However, the governance of phenotypic plasticity and photosynthetic performance by CBFs/DREBs in winter cultivars must also be integrated with the process of vernalization (Amasino, 2004; Sung and Amasino, 2005; Trevaskis et al., 2007; Trevaskis, 2010). Thus, gene regulation by *CBFs* extends well beyond its traditional role in cold acclimation and freezing tolerance. As discussed in detail by Murchie et al. (2009), elucidation of the mechanisms which govern the dynamic nature of energy partitioning between energetically “useful processes” involved in C and N-assimilation for biomass and seed production versus apparently energetically “wasteful processes” such as the dissipation of absorbed light energy as heat through NPQ to optimize plant survival remains a major challenge in maximizing crop productivity. Modulation of *CBF *expression levels may provide important new insights into potential molecular and genetic approaches focused on the maintenance or even the enhancement of plant productivity under suboptimal growth conditions associated with climate change (Dahal et al., 2012a).

## EXCITATION PRESSURE GOVERNS CHAOTIC LEAF VARIEGATION PATTERNS

The *A. thaliana* chaotic variegated mutant, *immutans (im)*, displays unpredictable white and green sectoring patterns due to differential stability of thylakoid membranes during chloroplast biogenesis and are considered plastid autonomous (Rodermel, 2002; Yu et al., 2007). IMMUTANS (IM) is encoded by a single nuclear gene which is translated into a 35 kD thylakoid polypeptide where it functions as a plastid terminal oxidase (PTOX). Cloning and characterization of *IM* revealed that it is related to the mitochondrial inner membrane AOX (Carol et al., 1999; Wu et al., 1999; McDonald et al., 2011) which oxidizes ubiquinol and reduces molecular oxygen (Vanlerberghe and McIntosh, 1997). By analogy, it has been suggested that IM is induced under stress conditions and oxidizes PQH_2_ and reduces O_2_ to water (Carol et al., 1999; Wu et al., 1999; Carol and Kuntz, 2001; Rodermel, 2001; McDonald et al., 2011). Consequently, PTOX is considered to be the essential oxidase which participates in the chlororespiratory pathway (Cournac et al., 2000a,b). Recently, Fu et al. (2012) reported that AOX1a and AOX2 can functionally substitute for PTOX in the *immutans* mutant of *A. thaliana* and rescue the variegated phenotype. It is proposed that IM is not only essential for carotenoid biosynthesis and chlororespiration but it is also acts as a “safety valve” in the photoprotection of PSII (Niyogi, 1999; Cournac et al., 2000a,b; Rodermel, 2001; Joet et al., 2002; Peltier and Cournac, 2002).

Recently, we reported specific growth conditions that completely suppressed the variegated phenotype of *im* such that it exhibited an “all green” phenotype indistinguishable from the wild type (WT) even though neither *IM* expression nor IM accumulation was detected (Rosso et al., 2006). By exploiting this phenomenon and comparing *im* knockout plants with WT, as well as a 6× and 16× over-expressor of *IM*, we reported that in mature, fully expanded leaves of *A. thaliana*, IM (PTOX) cannot compete with P700^+^ for PSII-generated electrons under optimal growth conditions. We concluded that under optimal growth conditions, PTOX cannot act as a simple “safety valve” in *A. thaliana* leaves exhibiting full photosynthetic competence (Rosso et al., 2006). Our conclusion is consistent with that of Heyno et al. (2009) who reported that over-expression of PTOX in tobacco induced rather than ameliorated oxidative stress. In contrast to these reports, there are numerous reports of specific abiotic stress conditions which induce the expression and accumulation of PTOX in alpine plant species (Streb et al., 2005), the halophile, *Thellungiella halophila* (Stepien and Johnson, 2009) as well as the marine cyanobacterium, *Synechococcus* WH8102 and marine green alga, *Ostreococcus* (Bailey et al., 2008; Cardol et al., 2008; Grossman et al., 2010). Furthermore, Baena-Gonzalez et al. (2003) reported that deletion of tobacco plastid *psbA* triggers an up-regulation of the thylakoid-associated NAD(P)H dehydrogenase complex as well as PTOX.

All oxygenic photoautotrophs exhibit the presence of IM (PTOX) in their genomes (McDonald et al., 2011). How can the apparent conflicting reports regarding the function of IM (PTOX) be reconciled? Meta-transcriptome analyses of PTOX indicated that PTOX expression is primarily developmentally regulated in *Arabidopsis* rather than by stress (Rosso et al., 2006). To address the role of IM (PTOX) in leaf development, we developed a sensitive, high resolution, non-destructive imaging technique by which we could quantify the extent of variegation as a function of time. This allowed us to quantify the effects of growth irradiance and temperature on the extent of variegation as a function of developmental time. Using this technique, we were able to show that the absence of IM is necessary but not sufficient to explain variegation in *A. thaliana*. In fact, the extent of variegation is governed by excitation pressure not only in *im* but also in the other *Arabidopsis* variegated mutants such as *spotty*, *var1,* and *var2* (Rosso et al., 2009). The biogenesis and assembly of thylakoid membranes requires tight co-ordination between the *de novo* synthesis of Chl and other pigments, lipids as well as chloroplast and nuclear encoded proteins (Eberhard et al., 2008; Sakamoto et al., 2008). This raises an important developmental question as to how a photoautotroph mitigates the potential damaging effects of photo-oxidative stress during the biogenesis and assembly of its photosystems prior to the establishment of a fully functional photosynthetic apparatus. In WT plants, protection from photo-oxidative stress is provided through transient stimulation of non-photochemical dissipation of excess energy through the xanthophyll cycle (Demmig-Adams and Adams, 1992; Murchie et al., 2009) as shown during early greening in barley (Krol et al., 1999) as well as the induction of myriad plant oxidative stress genes including *AOX* (Aluru et al., 2009). However, *im* seedlings are unable to biosynthesize photoprotective carotenoids involved in the xanthophyll cycle (Wetzel et al., 1994). Although IM cannot compete with P700^+^ for PSII-generated electrons in mature leaves that are photosynthetically competent (Rosso et al., 2006), its presence is essential to minimize excitation pressure and the potential for photo-oxidative damage during the very early stages in the assembly and biogenesis of the photosynthetic apparatus prior to the attainment of full photosynthetic competence (Rosso et al., 2006, 2009).

To account for the variable and unpredictable patterns of leaf variegation in *immutans*, we suggest the presence of a gradient of excitation pressure within the developing leaf primordia during light-dependent chloroplast biogenesis: the data indicate that if excitation pressure in a particular developing sector is lower than 0.2, chloroplast biogenesis proceeds normally and an “all green” sector(s) with a LEP phenotype will develop; however, if excitation pressure in a particular developing sector exceeds 0.2, thylakoid assembly and chloroplast biogenesis is inhibited and white sectors with a HEP phenotype will develop (Rosso et al., 2009). This implies a threshold-dependence for the sensor(s) that govern the HEP versus the LEP phenotype in *im* in response to changes in excitation pressure, and therefore, appear to act as “molecular on–off switches” in contrast to that observed for the regulation of the HEP phenotype in the green alga, *Chlorella vulgaris*.

There appears to be a consensus that PTOX not only acts as the terminal oxidase in the chlororespiratory pathway (Cournac et al., 2000a,b) but also acts an important alternative photosynthetic electron sink under any condition where PSI is acceptor-limited. Under these conditions, the induction of PTOX as an alternative, O_2_-dependent pathway for photosynthetic electron flow would mitigate any PSI limitation and protect PSII from over-excitation by oxidizing PQH_2_ and reducing O_2_ to water (Grossman et al., 2010; McDonald et al., 2011). This is consistent with the recent results of Formighieri et al. (2012) who reported that a *gun4* mutant of *Chlamydomonas reinhardtii* exhibits a significant decrease in PSI/PSII ratios coupled with an increase in PTOX activity which may protect the *gun4* mutant from HEP under conditions where PSI levels may be limiting. In addition, the biogenesis and assembly of the photosynthetic apparatus appears to be coordinated with mitochondrial redox balance as indicated by the fact AOX expression is modulated by excitation pressure originating within the chloroplast (Rosso et al., 2009) indicating redox communication between chloroplasts and mitochondria.

Unlike photoacclimation or LT acclimation discussed above, chaotic leaf variegation in *A. thaliana* is a consequence of destabilization of the developing photosynthetic apparatus by excitation pressure during thylakoid membrane assembly and chloroplast biogenesis rather than an example of remodeling of the photosynthetic apparatus by adjusting either σ_PSII_ or τ ^−1^. The control of variegation in various *Arabidopsis* mutants represents an excellent example of how chloroplast redox sensing/signaling, through excitation pressure, mediates the interaction of the nuclear encoded *IMMUTANS* gene with its environment to affect chloroplast biogenesis, leaf development, and subsequent phenotype.

## WHAT IS THE PRIMARY SITE(S) FOR SENSING CHLOROPLAST ENERGY IMBALANCE?

Research with cyanobacteria (Fujita, 1997), green algae (Escoubas et al., 1995; Maxwell et al., 1995a,b; Hüner et al., 1998; Wilson et al., 2003), and plants (Anderson et al., 1995; Pfannschmidt, 2003; Fey et al., 2005; Woodson and Chory, 2008; Brautigam et al., 2009; Pesaresi et al., 2009; Rochaix, 2011) indicates that a key component of redox sensing/signaling associated with the photosynthetic apparatus is the PQ pool, a mobile electron carrier that shuttles electrons from PSII to the Cyt b_6_/f. This is based, in part, on experiments where the characteristic, yellow-green, HL phenotype of green algae brought about by photoacclimation, that is, acclimation to high irradiance, could be mimicked by chemically modulating the redox status of the intersystem PQ pool. This is traditionally accomplished by using minimal concentrations of the PET inhibitors, either 2,5-dibromo-3-methyl-6-isopropylbenzoquinone (DBMIB) or 3-(3′,4′-dichlorophenyl)-1,1-dimethylurea (DCMU; Escoubas et al., 1995; Pfannschmidt et al., 1999; Pfannschmidt, 2003; Wilson et al., 2003; Piippo et al., 2006). Since DBMIB inhibits the oxidation of PQH_2_ by the Cyt b_6_/f complex, PSII keeps the PQ pool reduced in the light. This induces the HL phenotype which is characterized by low Chl content per cell, high Chl a/b ratio (>10), accumulation of the carotenoid-binding protein (Cbr; Krol et al., 1997) but suppression of both *Lhcb2 *expression and Lhcb2 accumulation through retrograde regulation of nuclear encoded genes by the chloroplast in *Chlorella vulgaris* and *D. salina*. Since this HL phenotype is also mimicked by growth of *Chlorella vulgaris* and *D. salina* at LT (Maxwell et al., 1995a,b; Wilson et al., 2006), we designate this as a HEP phenotype. In contrast, since DCMU prevents the exit of electrons from PSII reaction centers into the PQ pool, PSI is able to keep the PQ pool oxidized in the light. Under these conditions, cells exhibit a LEP phenotype characterized by a high Chl content per cell, low Chl a/b ratio (3.0–3.5), and high levels of *Lhcb2* expression and Lhcb2 accumulation (Escoubas et al., 1995; Maxwell et al., 1995a,b; Wilson et al., 2003). This LEP phenotype can also be generated by growth at either low irradiance or high temperature but moderate irradiance in *Chlorella vulgaris* (Maxwell et al., 1995a,b; Hüner et al., 1998; Wilson et al., 2003). Since Q_A_, the first stable quinone electron acceptor within the PSII reaction center is considered to be in rapid equilibrium with the PQ pool of intersystem electron transport (Dietz et al., 1985; Schreiber et al., 1994; Maxwell et al., 1995a,b; Baker, 2008), we have assumed that the PQ pool is the primary redox sensor that governs changes in excitation pressure (Hüner et al., 1998, 2003, 2006; Oquist and Huner, 2003; Ensminger et al., 2006; Morgan-Kiss et al., 2006; Wilson et al., 2006; McDonald et al., 2011). However, our earlier report for the regulation of HEP and LEP phenotypes in the filamentous cyanobacterium, *P. boryanum*, indicated that the PQ pool is probably not the major site from which redox signals emanate to control pigmented phenotype through modulation of the composition and structure of phycobilisomes in this cyanobacterium (Miskiewicz et al., 2000, 2002). This is consistent with the recent report by Piippo et al. (2006) who reported that the PQ pool is not the major redox sensor regulating photoacclimation in *A. thaliana* and that the primary redox signals probably emanate from the acceptor-side of PSI.

The assumption that the combination of DCMU and DBMIB identifies the redox state of the PQ pool as the primary site of chloroplast redox sensing/signaling ignores the potential contributions of other PET components to redox sensing/signaling. Indeed, recent research in *Arabidopsis *suggests that the redox state of ferredoxin (Fd), thioredoxins (Trx), and peroxiredoxins on the acceptor-side of PSI (Dietz, 2003, 2008; Dietz and Scheibe, 2004) as well as the generation of reactive oxygen species (ROS; Apel and Hirt, 2004; Wagner et al., 2004) may constitute a complex network of redox senors/signals involved in the retrograde pathway of communication from the chloroplast to the nucleus (Koussevitzky et al., 2007; Fernandez and Strand, 2008; Jung and Chory, 2010). In fact, Piippo et al. (2006) suggest that the reducing side of PSI may represent the major source of chloroplast redox signaling involved in retrograde regulation. Furthermore, PSII itself may also contribute to retrograde regulation of nuclear genes through the generation of singlet oxygen (Apel and Hirt, 2004; Wagner et al., 2004; Nott et al., 2006; Lee et al., 2007; Fernandez and Strand, 2008) and thus, may contribute to redox regulation by excitation pressure. Using a combination of inhibitors, uncouplers, and antimycin A, Chen et al. (2004) identified two different sensors involved in the retrograde signal transduction pathway in *D. salina. *The transthylakoid membrane potential (pmf) appeared to govern gene expression in response to changes in irradiance on a short time scale (<4 h), whereas on time scales of 8 h or longer, the redox state of the PQ pool appeared to become the more prominent sensor. Thus, the regulation of gene expression and phenotypic plasticity through excitation pressure in green algae must represent a complex interacting *intracellular *network of sensors and signal transduction pathways.

However, in terrestrial crop plants and *A. thaliana*, a similar complex, intracellular network must be integrated with an equally complex sensor/signal transduction pathway that extends over long distances from leaf chloroplasts to meristematic tissue such as the crown in cereals. This long distance sensor/signaling pathway must convey information regarding the redox status of the leaf chloroplasts to regulate meristematic cell division and differentiation and affect plant growth, development, and morphology (Gray et al., 1997). There is a growing body of evidence that the environment has a significant impact on cell development not only in leaves (Rosso et al., 2009) but also in roots (Tsukagoshi et al., 2010) as well as anthers (Kelliher and Walbot, 2012; Whipple, 2012) through modulation of cellular redox state. Furthermore, there is growing support in the literature that although hydrogen peroxide is a toxic molecule generated by various stress conditions in plants (Mittler et al., 2004), it is also an important molecule involved in systemic signaling and acclimation to excitation pressure in plants (Karpinski et al., 1999; Mullineaux et al., 2000; Fryer et al., 2003; Mullineaux et al., 2006; Geisler et al., 2006).

## THE “GRAND DESIGN OF PHOTOSYNTHESIS”

**FIGURE 2:**
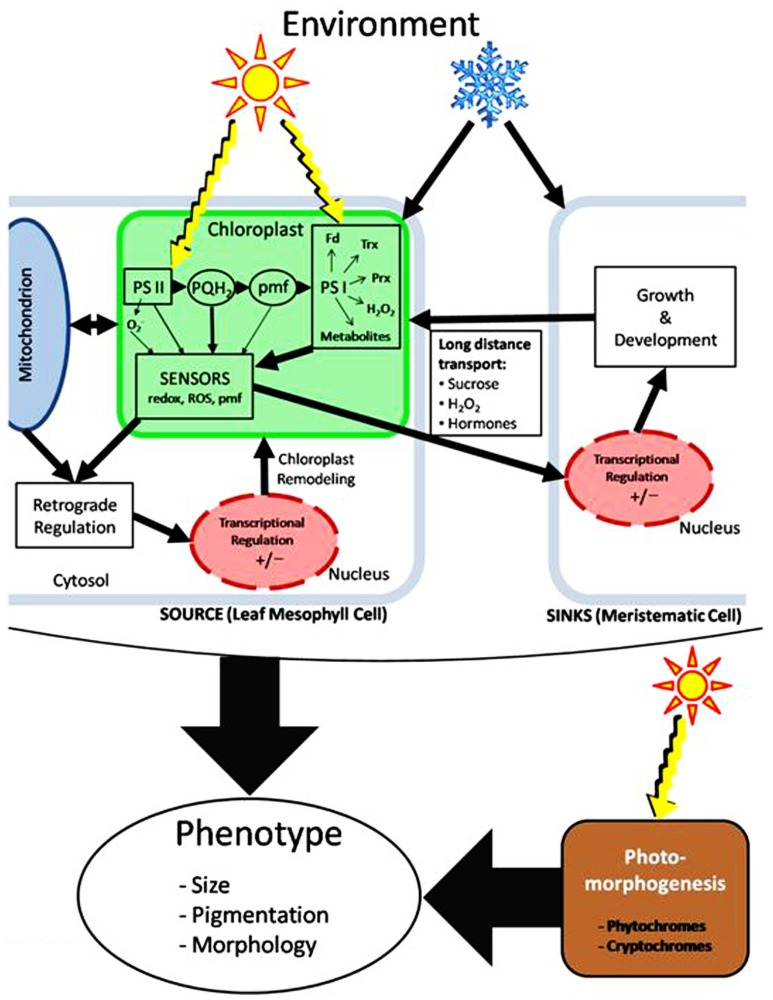
**A model illustrating the environmental regulation of phenotypic plasticity via chloroplast redox sensing coupled to both intracellular and long distance signal transduction.** Light is the ultimate source of energy for photoautotrophs. Energy imbalances generated either by changes in high light (sun) or low temperature (snowflake) are sensed in the chloroplast by modulation of the redox state of the photosynthetic apparatus. Such redox information is exchanged between chloroplasts and mitochondria through carbon metabolism. Intracellular redox imbalances in chloroplasts and mitochondria are conveyed to the nucleus through retrograde regulation to affect remodeling of the photosynthetic apparatus to re-establish photostasis and energy balance. The remodeling of the photosynthetic apparatus may be reflected in changes phenotype exhibited as alterations in pigmentation in certain algae and cyanobacteria. In vascular plants, information regarding chloroplast redox imbalance is also conveyed to distant sinks such as meristematic regions of a plant to affect growth and development. This occurs through long distance signaling via the vascular system. Such information also contributes to the regulation of phenotypic plasticity reflected in changes in vascular plant morphology. In plants, distant sink limitations induced by low temperature can also be important in feedback regulation of energy balance in chloroplasts in source leaves. Thus, phenotypic plasticity is the result of the integration of both photosynthetic as well as photomorphogenic events. PSII, photosystem II; PSI, photosystem I; PQH_2_, reduced plastoquinone; pmf, proton motive force; Fd, ferredoxin; Trx, thioredoxins; Prx, peroxiredoxins.

**Figure 2** represents a simplified model that attempts to summarize the central role of the photosynthetic apparatus as a major sensor of excitation pressure that is modulated by irradiance (sunlight) and LT (snowflake) in photoautotrophs. It is clearly established that the PET chain is an important source of redox signals involved in intracellular redox signaling. There appears to be a consensus that, within the PET chain, several potential redox sensors appear to exist including PSII, the PQ pool, the thylakoid proton motive force (pmf) as well as the acceptor-side of PSI. The latter would include the redox status of Fd, Trx, peroxiredoxins, H_2_O_2_, as well as metabolic intermediates of carbon metabolism. The acceptor-side of PSI appears to be a major site of redox sensing/signaling in the chloroplast. These sensors may contribute to varying extents and may act independently or in concert to initiate retrograde signaling to the nucleus to affect transcriptional regulation of nuclear photosynthetic genes. The end result of such retrograde redox sensing/signaling in response to changes in excitation pressure is the remodeling of the structure and function of the photosynthetic apparatus to re-establish photostasis.

In addition to intracellular retrograde redox sensing/signaling, redox signals from the chloroplast must be transmitted to various meristematic regions such as the crown tissue within winter cereals to affect plant morphology (e.g., dwarf phenotype). Consequently, this redox signaling pathway probably involves long distance transport via the plant vascular system. Possible components of this long distance signaling pathway may include but are not restricted to hormones, H_2_O_2_, and photosynthetic end-products such as sucrose. The rate of long distance transport from source to sink as well as sink activity reflected in rates of growth and development can, in turn, feedback regulate the extent of chloroplastic excitation pressure. Thus, we suggest that phenotypic plasticity governed by excitation pressure initially sensed in leaf chloroplasts is the result not only of local remodeling of the photosynthetic apparatus, but also the regulation of remote meristematic regions to affect plant morphology.

Thirty years ago, Arnon (1982) proposed the concept of a “grand design of photosynthesis” which was re-introduced by Anderson et al. (1995). Anderson et al. (1995) conclude that the “grand design of photosynthesis with exquisite regulation ensures that the responses of both photoreceptors and photosystems II and I, acting as their own light sensors, are inextricably linked with feedback metabolic responses from photosynthesis itself, which allow plants to respond to both sudden and sustained fluctuations in environmental cues.” This notion has been supported either directly (Anderson et al., 1995; Hüner et al., 1998; Ensminger et al., 2006; Wilson et al., 2006; Pfannschmidt and Yang, 2012) or indirectly (Pfannschmidt, 2003; Fey et al., 2005; Murchie et al., 2009; Lepisto and Rintamaki, 2012). Although the precise nature of the redox sensors and signal transduction pathways associated with excitation pressure remain to be elucidated, we maintain that the data summarized in this review are consistent with the notion of a “grand design of photosynthesis.” Consequently, we suggest that, in addition to its traditional role as the energy transformer for the biosphere, the photosynthetic apparatus should also be considered a major energy sensor which is modulated by environmental cues and plays a major role in the regulation of phenotypic plasticity (**Figure 2**).

Although all photoautotrophs can sense changes in their environment through the modulation of excitation pressure, we suggest that it is source–sink relationships that ultimately modulate the extent of excitation pressure which then governs the observed phenotype of photoautotrophs during growth at either HL or LT. Thus, photoautotrophs must integrate information regarding changes in light quality through photoreceptors with changes in light as an energy source through the redox state of the photosynthetic apparatus to affect plant growth and morphology (Anderson et al., 1995; Fryer et al., 2003; Lepisto and Rintamaki, 2012; **Figure 2**). Since sunlight represents the ultimate energy source for our biosphere, greater understanding of the role of the chloroplast as an energy sensor which governs energy partitioning and energy utilization efficiency as well as phenotypic plasticity will be essential for the maintenance or possibly even the enhancement of plant biomass for energy and crop productivity for food under suboptimal growth conditions associated with climate change (Murchie et al., 2009; Dahal et al., 2012a).

## Conflict of Interest Statement

The authors declare that the research was conducted in the absence of any commercial or financial relationships that could be construed as a potential conflict of interest.
